# Notes from the June Meeting of the American Medical Asssociation

**Published:** 1887-06

**Authors:** 


### NOTES FROM THE JUNE MEETING AMERICAN MEDICAL ASSOCIATION.

Dr. A. Y. P. Garnett of Washington, was elected President. Dr. Duncan Eve, of Nashville, Vice-President.

Dr. R. W. Park, of Waco, represented Texas on the Nominating Committee and is on the Committee on Necrology.

Next place of meeting, Cincinnati, 4th Tuesday in May, 1888.

This Convention, according to the *Medical Standard*, adopted amendments to the Constitution, on the jump:—without letting them lie over one year, as required by the Constitution. The *Standard* says: "the President was unequal to the emergency, and made a mistake in his ruling on the subject"—quite a common thing with Presidents of Medical Associations, by the bye.

Fifteen hundred delegates and members attended the meeting. By the bye, delegates, only, are *entitled* to vote, according to the Constitution and By-laws. So pointed out by Dr. Davis; but it seems that every body voted, as they usually do in medical associations.

"Dr. Dudley S. Reynolds of Louisville, is the champion speaker, smoker and story-teller of the Convention, and withall, one of its strongest "pillars." Few men are so magnificently endowed as to personal presence, heroic voice, and clear comprehension, as is the "lone fisherman" whose editorial motto is "Progress.""—*Medical Standard.*

For enterprise, commend us to the *Medical Standard.* It got out a handsome daily edition during the four days session of the American Medical Association, in neat pamphlet cover. No. 3, contains a cut of Dr. N. S. Davis, the "father," anc No. 4, very good likeness of the irrepressible Shoemaker, the indomnitable Atkinson, the enterprising Love, the energetic Porter, the superb Wile, the indefatigable Wathen, Fenger, Buford, Spitzka and others. The only thing we regret about it, is that the *Standard* did not include the "champion speaker and smoker," Dudley S. Reynolds. — the "progressive !" "Ninety Cataract Operations under Cocaine without a failure," is a record of which any man may be proud.

Dr. Wm. Porter of St. Louis was elected President of Association of American Medical Editors, andDr. W. C. Wile, of Philadelphia, Secretary.

President Gregory's Address was on "cell antagonism." The style was decidedly laconic; here's a sample; the opening paragraph:

"Obviously, force and matter "make up" the universe; force implies antagonism; antagonism perpetuates motion. The living cell is the embodiment of nature. Cell antagonism is life. Multicellular organisms represent a community of vital unities. A model organism in equilibrium is health. Cell struggle is the gist of modern pathology. Every element and every organ are (?) vulnerable" etc.

It has been pronounced "able."

The American Medical Association, appropriated $1000.00 for the International Medical Congress. Illinois State Medical Association gave $750.00.

